# Protoporphyrin IX plasma and blood pharmacokinetics and brain tumor distribution determined by a validated LC–MS/MS method

**DOI:** 10.1038/s41598-025-05780-w

**Published:** 2025-07-01

**Authors:** William Knight, Tigran Margaryan, Kamal Shaik, Nader Sanai, Artak Tovmasyan

**Affiliations:** Ivy Brain Tumor Center, Barrow Neurological Institute, St. Joseph’s Hospital & Medical Center, 350 West Thomas Road, NRC 403, Phoenix, AZ 85013-4496 USA

**Keywords:** Protoporphyrin IX (PPIX), Liquid chromatography tandem mass spectrometry (LC–MS/MS), 5-aminolevulinic acid (5-ALA), Sonodynamic therapy, Brain tumor, Photodynamic therapy, Bioanalytical chemistry, Mass spectrometry, Surgical oncology, CNS cancer, Predictive markers, Diagnostic markers, CNS cancer

## Abstract

Protoporphyrin IX (PPIX) is a fluorescent metabolite in the heme biosynthesis pathway, and cancer cells accumulate it when 5-aminolevulinic acid (5-ALA), a precursor, is administered. In the U.S., 5-ALA is approved for visualizing high-grade gliomas (HGG) during fluorescence-guided surgery. PPIX is also central to experimental photodynamic and sonodynamic therapies for HGG. Additionally, PPIX measurement is critical for diagnosing and monitoring porphyrias. Despite the need for a sensitive and rapid bioanalytical method for accurate quantification of PPIX in biospecimens, no reliable validation of an LC–MS/MS method is available due to challenges related to its chemical instability, poor solubility, and tendency to aggregate. This work is the first to present a fully validated, sensitive, and rapid LC–MS/MS method for determining PPIX levels in human plasma, blood, and brain tumors. The method overcomes stability concerns, achieving a 3.5-min total run-time with a concentration range of 1–2000 nmol/L for plasma and tumors, and 10–2000 nmol/L for blood. Application of the method in a clinical trial, which assesses sonodynamic therapy for HGG patients, shows significant PPIX production, peaking in plasma and blood six hours post-5-ALA administration. In recurrent HGG patients, PPIX levels were notably higher in gadolinium-enhancing tumor regions compared to non-enhancing areas, indicating preferential accumulation in tumors.

## Introduction

Protoporphyrin IX (PPIX) is a fundamental intermediate in the heme biosynthesis pathway with significant roles in various medical applications due to its fluorescent properties, ability to accumulate in tumor cells, and capacity to generate reactive oxygen species (ROS) when exposed to light or ultrasound^1^. These applications are primarily mediated through the administration of 5-aminolevulinic acid (5-ALA), a precursor in PPIX biosynthesis^2^.

In cancer diagnostics, 5-ALA-induced PPIX fluorescence is utilized in fluorescence-guided surgery (FGS), where the fluorescence of PPIX helps surgeons visualize high-grade gliomas (HGG), including glioblastoma (GBM)^3^. This enhanced visualization is crucial for distinguishing malignant from healthy tissues, thereby improving the accuracy of tumor resection. Gleolan, a commercial form of 5-ALA, has been approved in the United States specifically for use in GBM surgery^4^.

Beyond surgery, PPIX plays a pivotal role in photodynamic therapy (PDT). In this treatment modality, PPIX acts as a photosensitizer. When activated by light, PPIX generates ROS, which induce oxidative stress and apoptosis in cancer cells^5^. This method is particularly effective for treating superficial tumors and other superficial lesions, warranting its current investigation and use for treating a variety of cancers and skin conditions^6–9^. The selective accumulation of PPIX in cancer cells following 5-ALA administration enhances the specificity and efficacy of PDT, which is driven by differential expression of biosynthetic enzymes and transporters between cancer and normal cells^5,6,10–12^. PPIX is also used as a sonosensitizer in the experimental therapy known as sonodynamic therapy (SDT)^13^. SDT involves the use of focused ultrasound and PPIX to initiate cancer cell death. Although mechanisms are not fully understood, one hypothesis behind SDT includes the phenomenon of sonoluminescence, whereby ultrasound-induced cavitation generates light that activates PPIX^14^. This novel approach holds promise for non-invasive treatments, especially for deep tissue tumors^15,16^.

Elevated levels of PPIX after 5-ALA administration have also been investigated as potential diagnostic markers for various cancers and metabolic disorders^17–20^. This selective accumulation in abnormal cells makes PPIX a valuable biomarker for early cancer detection and monitoring of treatment efficacy^17–20^. Notably, several studies have demonstrated the diagnostic value of PPIX in HGG^20^, bladder cancer^17^, and pancreatic cancer^21^, highlighting its broader clinical relevance across tumor types. PPIX also plays a crucial role in the context of porphyrias, a group of disorders caused by abnormalities in the heme biosynthesis pathway^22^. In certain types of porphyria, such as erythropoietic protoporphyria and X-linked protoporphyria, elevated PPIX levels are observed in blood and other tissues^22^. This accumulation results in severe photosensitivity, where exposure to sunlight causes painful skin reactions, swelling, and potential long-term damage^23^.

Given the significant importance of PPIX in medicine, quantification of its levels in biological matrices is crucial for treatment, diagnosis, and management of various diseases. Several bioanalytical methods have been reported for quantification of PPIX levels in biological matrices. These methods are mostly based on high performance liquid chromatography (HPLC) coupled with photodiode array and fluorescence detection as well as spectrofluorometry^19,24–27^. However, the available methods have low specificity and sensitivity, laborious sample processing, and long analytical run time.

In this article, a novel, rapid, and sensitive liquid chromatography tandem mass spectrometry (LC–MS/MS) method was developed and fully validated according to the guideline published by the International Council for Harmonisation (ICH)^28,29^, which are followed by the United States Food & Drug Administration (U.S. FDA) and European Medicines Agency (EMA). Several LC–MS/MS methods were described in the literature that assess PPIX levels in various biomatrices, including serum, bacteria, and plants^20,25,30–35^, yet none of them were validated. A publication by Novak et al. has reported two LC–MS-based bioanalytical methods that were used to determine PPIX levels in patients’ plasma^36^. While the methods were validated to be used in the associated clinical trials in patients with actinic keratosis, the major focus of the manuscript was to report clinical pharmacokinetics and safety data of a 5-ALA formulation, and most of the validation test components related to PPIX determination in plasma were not reported. In this respect, this manuscript is the first to report a fully validated, rapid LC-MS/MS method with superior sensitivity compared to existing methods, specifically developed for use with human plasma, blood, and brain tumor. The present method has minimal sample preparation, lower limit of quantitation of 1 nM, and total analytical run time of 3.5 min. During the development and validation of the method, a few challenges related to analyte stability in various matrices were addressed. The bioanalytical method is currently employed in a 5-ALA SDT clinical trial (NCT04559685) to determine the pharmacokinetic profile in plasma and blood of recurrent HGG patients. The method is also used to assess PPIX levels in the brain tumors of HGG patients undergoing FGS, with the aim of optimizing SDT treatment parameters. While the initial application presented here involves small patient cohorts for SDT (n = 3) and FGS (n = 4), these results are intended to illustrate feasibility and translational applicability of the validated method. The associated clinical studies are ongoing, and future analyses will incorporate a more comprehensive dataset encompassing full pharmacokinetic and pharmacodynamic profiles, as well as safety and efficacy endpoints. Importantly, this validated bioanalytical method may also support future investigations into the utility of plasma PPIX concentrations as a potential biomarker for GBM and other malignancies.

## Materials and methods

### Chemicals and reagents

**Fig. 1:**
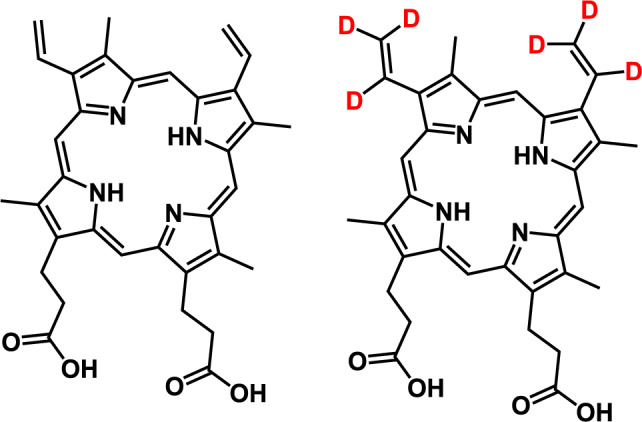
Chemical structure of PPIX and internal standard, ^2^H_6_-PPIX.

Protoporphyrin IX (purity 97.8%) (Fig. 1) was obtained from Enzo Life Sciences (Farmingdale, New York, USA). A stable isotope labeled form of PPIX (^2^H_6_-protoporphyrin IX, purity 97%) (Fig. 1) was purchased from AlsaChim SAS (Illkirch, France) and was utilized as an internal standard (IS). LC–MS grade water was obtained from Milli-Q IQ 7000 Filter water system (MilliporeSigma, Burlington, Massachusetts, USA). Ammonium formate and formic acid were purchased from Fisher Scientific (Hampton, New Hampshire, USA). Dimethyl sulfoxide (DMSO) was purchased from MilliporeSigma and *N*,*N*-dimethylformamide (DMF) was purchased from Sigma-Aldrich (St. Louis, Missouri, USA). Methanol and acetonitrile were purchased from VWR (Radnor, Pennsylvania, USA). Human plasma (anticoagulant: K_2_EDTA) was purchased from BioChemed (Winchester, Virginia, USA) and Innovative Research, Inc. (Novi, Michigan, USA). Human whole blood was purchased from Innovative Research, Inc. Human brain was received from VRL Eurofins (Denver, Colorado, USA).

### Instrumentation and chromatographic conditions

**Fig. 2:**
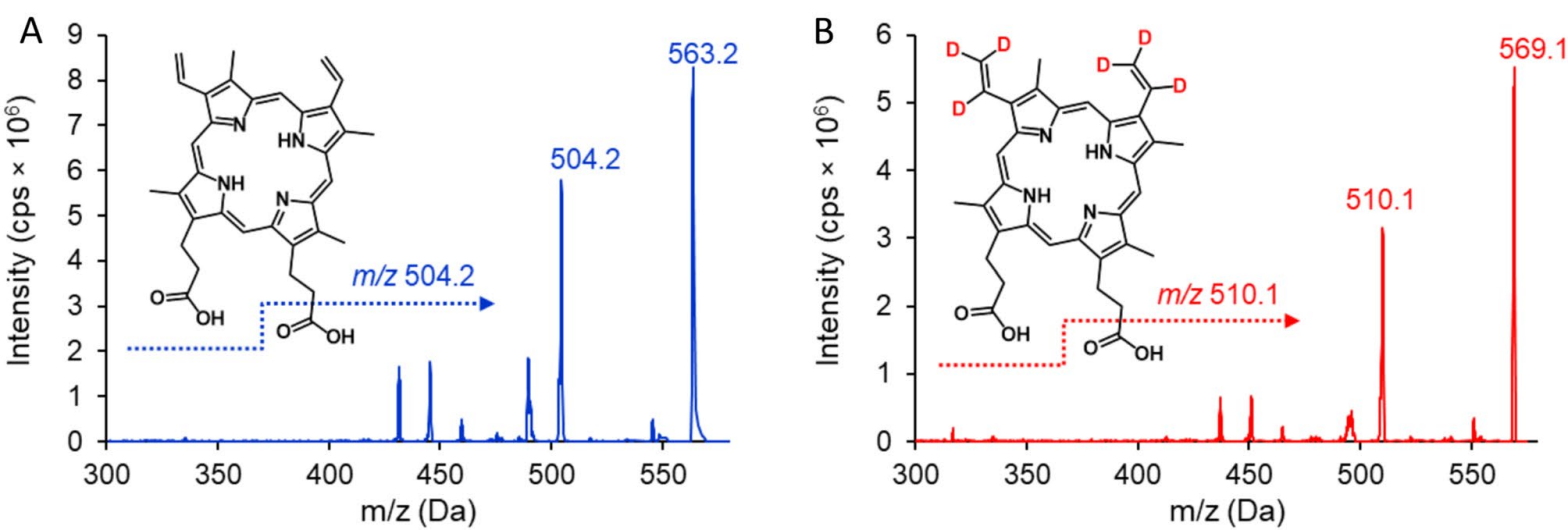
MS/MS fragmentation and representative product ion scan spectra of PPIX and internal standard, ^2^H_6_-PPIX.

PPIX levels in plasma, brain homogenate, and whole blood samples were determined using a Sciex ExionLC UHPLC system coupled with a Sciex Triple Quad 6500+ detector (Foster City, CA, USA) equipped with electrospray ionization (ESI). Chromatographic separation was achieved using Phenomenex Kinetex™ EVO C18 (50 × 2.1 mm, 1.7 µm) column, with column oven temperature set to 40°C. With autosampler temperature set to 5°C, 3 µL were injected into the system with mobile phases (A) 5 mM ammonium formate containing 0.1% formic acid and (B) 1/1 acetonitrile/methanol containing 0.1% formic acid. Samples were detected with isocratic elution with a total run time of 3.5 min and flow rate of 0.5 mL min^−1^. Mass spectrometry source parameters were set to the following: ion source voltage was 5500 V, source temperature was 500 °C, curtain gas, nebulizing gas, and heating gas were 30, 80, and 60 psi respectively. PPIX and IS mass transitions (m/z 563.2 → 504.2 and 569.1 → 510.1, respectively), determined in multiple reaction monitoring mode, were optimized with DP of 100 V, EP of 10 V, CXP of 27 V, and collision energy of 56 V with collision gas held at 9 psi (Table 1, Fig. 2). The dwell time for each mass transition was set to 200 ms. Data acquisition and analysis were performed using Analyst software version 1.7.

**Table 1:** MS parameters.

Parameters	Value
MRM* transition (standard), m/z	563.2 → 504.2
MRM transition (internal standard), m/z	569.1 → 510.1
Declustering potential, V	100
Entrance potential, V	10
Collision energy (standard), V	56
Collision energy (internal standard), V	56
Collision cell exit potential, V	27
Curtain Gas, psi	30
Collision Gas, psi	9
IonSpray Voltage, V	5500
Temperature, °C	500
Ion Source Gas 1 (nebulizing), psi	80
Ion Source Gas 2 (heating), psi	60

*MRM—multiple reaction monitoring.

### Sample preparation

#### Calibration standards and quality control samples

PPIX stock solutions were prepared at a concentration of 1 mM and dissolved in DMF. PPIX IS stock solution was dissolved at 2 mM due to the low quantity available and dissolved in DMSO. Calibration curve (CC) and quality control (QC) working solutions were prepared by diluting stock solutions with methanol. Two separate stock solutions were prepared and compared with each other to confirm an accurate weighing, then combined in equal amounts. The combined stock was used to prepare both CC and QC working solutions. The IS precipitation solution (IS-PS, 20 nM ^2^H_6_-PPIX) was prepared by diluting the IS stock solution with methanol. CC standards were prepared by spiking blank human plasma with the appropriate working solution in a 1:19 ratio. The plasma batches received from the vendors had PPIX levels below LLOQ. However, it is advised to deplete the PPIX levels via light exposure (> 1500 lx) for at least 1 h when preparing CC and QC standards if the batches contain endogenous PPIX levels above LLOQ. QC standards for plasma and brain homogenate were also prepared by spiking with working solutions in a 1:19 ratio. For whole blood QCs, the preparation of the lower limit of quantitation QC (LLOQ QC) involved a dilution of whole blood with blank plasma to reach the LLOQ of the assay, accounting for the endogenous levels of PPIX in the specific lot of blood used. The rest of the QCs were spiked with working solutions in varying amounts to reach the desired concentration, accounting for endogenous PPIX. The analyte concentrations in the calibration curve in human plasma, used for PPIX quantitation in plasma and brain homogenate samples, were 1, 2.5, 10, 20, 50, 100, 200, 500, 1000, and 2000 nM. For PPIX quantitation in blood samples, a surrogate plasma calibration curve was used with analyte concentrations of 10, 20, 50, 100, 200, 500, 1000, and 2000 nM. Five QC levels were used during validation: LLOQ QC, low (LQC), medium (MQC), high-medium (HMQC), and high (HQC). QC concentrations in plasma and brain homogenate were 1 nM (LLOQ QC), 3 nM (LQC), 60 nM (MQC), 600 nM (HMQC), and 1500 nM (HQC). QC concentrations in blood were 10 nM (LLOQ QC), 30 nM (LQC), 62.9 nM (MQC), 624 nM (HMQC), and 1520 (HQC). Stock solutions were stored at 4 °C while CC, QC, and working solutions were stored at − 80 ± 15 °C.

#### Plasma sample preparation

Plasma samples were thawed at room temperature. Once samples had thawed, a 50 µL aliquot was transferred to a 1.5 mL microcentrifuge tube. The sample was precipitated with 450 µL of IS-PS and agitated with a vortex mixer for at least 10 s, followed by centrifugation for 10 min at 12000 rpm and 4 °C. 100 µL of the supernatant was diluted with 600 µL of methanol in a glass autosampler vial. 3 µL of the resulting solution was injected into the LC–MS/MS. All steps in the sample preparation method until protein precipitation were performed under low light (< 5 lx) conditions.

#### Brain and brain tumor sample preparation

Blank brain homogenate was prepared by homogenizing brain tissue with water (1:3, mass (mg):volume (µL)) in bulk with a digital probe homogenizer (PRO25D, Pro Scientific, USA). To degrade any endogenous PPIX present in the brain tissue, the resultant homogenate was left on the bench exposed to light (> 1500 lx) for at least 1 h. Next, the 1:3 homogenate was diluted in a 1:1 ratio with an aqueous solution containing 10 mM ammonium formate and 0.4% formic acid. The resulting 1:7 acidified brain homogenate was mixed well before use. All brain QC samples were prepared using the 1:7 acidified brain homogenate. For the homogenization of clinical brain tumor samples, samples were homogenized with 5 mM ammonium formate containing 0.2% formic acid (1:7) at 6.00 m/s speed for 40 s with three cycles by Bead Ruptor Elite homogenizer (Omni International, USA).

Brain and brain tumor homogenate samples were thawed at room temperature. Once samples had thawed, a 50 µL aliquot was transferred to a 1.5 mL microcentrifuge tube. The sample was precipitated with 450 µL of IS-PS and agitated with a vortex mixer for at least 10 s, followed by centrifugation for 10 min at 12000 rpm and 4 °C. 100 µL of the supernatant was diluted with 600 µL of methanol in a glass autosampler vial. 3 µL of the resulting solution was injected into the LC–MS/MS. All steps in the sample preparation method until protein precipitation were performed under low light (< 5 lx) conditions.

#### Blood sample and surrogate plasma CC and QC preparation

Blood samples were thawed at room temperature. Once samples had thawed, a 10 µL aliquot of blood was transferred to a 1.5 mL microcentrifuge tube containing 40 µL of water. The 10 µL blood aliquot was spiked directly into the water and the resulting mixture was immediately and briefly vortex mixed. The sample was precipitated with 450 µL of IS-PS and agitated with a vortex mixer for at least 10 s, followed by centrifugation for 10 min at 12000 rpm and 4 °C. 100 µL of the supernatant was diluted with 600 µL of methanol in a glass autosampler vial. 3 µL of the resulting solution was injected into the LC–MS/MS. All steps in the sample preparation method until protein precipitation were performed under low light (< 5 lx) conditions.

For preparation of the surrogate matrix CC and QC samples, plasma was used. Plasma samples were thawed at room temperature. Once samples had thawed, a 10 µL aliquot was transferred to a 1.5 mL microcentrifuge tube. Next, 40 µL of water were added to the tube. The sample was briefly mixed and precipitated with 450 µL of IS-PS and agitated with a vortex mixer for at least 10 s, followed by centrifugation for 10 min at 12000 rpm and 4 °C. 100 µL of the supernatant was diluted with 600 µL of methanol in a glass autosampler vial. 3 µL of the resulting solution was injected into the LC–MS/MS. All steps in the sample preparation method until protein precipitation were performed under low light (< 5 lx) conditions.

### Method validation

Complete method validation for plasma, brain homogenate, and blood was performed to evaluate the following: sensitivity, selectivity, precision, accuracy, linearity, carryover, matrix effect, and various stability tests. The hemolysis effect could not be assessed due the nature of the endogenous analyte. All method validation tests were performed according to the ICH M10 guideline for bioanalytical method validation adopted by the U.S. FDA and EMA for bioanalytical method validation^28^.

#### Sensitivity and selectivity

Six different lots of single donor human plasma were screened for possible interferences at the retention time of the analyte and IS. One lot of control human brain tissue was also screened. Six different lots of single donor human blood were also screened for possible interferences at the retention time of the IS. The interference arising from the blank plasma and brain homogenate samples was compared to the peak area of the analyte and IS in the LLOQ sample. To calculate interference at the retention time of the IS in blood matrices, the interference arising from the blank was compared to the minimum IS response in the same run.

Any possible interference arising from the analyte at the retention time of the IS was determined by injecting the highest standard concentration without the IS in triplicate. To determine any interference arising from the IS at the retention time of the analyte, an extracted solution of the IS prepared without the analytes was injected in triplicate.

#### Calibration curve, linearity, precision, accuracy, and carryover

The calibration curve consisted of ten standards spiked into blank human plasma at the following concentrations: 1, 2.5, 10, 20, 50, 100, 200, 500, 1000, and 2000 nM. A blank (spiked with IS only) and double blank (neither IS nor analyte) were included as part of the curve. Linear regression with a weighting factor of 1/x^2^ was applied to describe the concentration–response relationship. The same regression and weighting factor were also applied to the calibration curve for blood quantitation, which was a truncated version of the original curve (10–2000 nM).

Carryover was evaluated by injecting double blank samples after an injection of various concentrated standard samples (500, 1000, and 2000 nM). Six replicates of double blank samples were injected after the ULOQ to determine the maximum number of blank injections necessary to achieve < 20.0% of LLOQ levels. As such, the peak area at the retention time of the analyte was calculated with an acceptance criterion of no more than 20.0% of LLOQ peak area. The acceptance criterion for IS carryover was set to < 5.0% of the average IS responses of the accepted calibrators and QCs at the IS retention time.

#### Matrix effect

The matrix effect was evaluated using six different lots of single donor human plasma, six different lots of single donor human blood, and one lot of human brain homogenate. The matrix effect was evaluated by preparing low and high QC samples in each matrix lot. Acceptance criteria for the evaluation were such that the accuracy should be ± 15.0% of the nominal value at each level, and that the coefficient of variance (CV, %) should not be greater than 15.0%.

In the six different blood lots included in the test, the endogenous levels present were also determined during the run. The amount added from spiking either LQC or HQC was added to the endogenous level which was then considered the nominal value.

#### Parallelism

Parallelism of the assay was assessed by preparing a series of diluted samples with the surrogate matrix. For example, brain homogenate and blood samples were diluted with blank plasma in ratios of 1:1, 1:4, 1:9 and 1:19 and analyzed with their respective surrogate calibration curves. The acceptance criterion to demonstrate parallelism was a percent CV of no more than 30% when comparing the diluted samples with the original sample.

#### Recovery

Recovery of PPIX from plasma, brain homogenate, and blood samples was assessed by comparing the mean area ratio of analyte peak to IS peak from five replicates of three QC levels (low, high-medium, and high) to the mean area ratio from five replicates of post-extraction spiked samples. To compensate for variations in analysis due to its sensitive nature, IS was spiked into each sample post-extraction. The same process was repeated for the IS to determine IS recovery. PPIX was spiked into each sample after extraction to compensate for variation in analysis by determination of area ratio. Acceptance criteria for both IS and analyte (low, high-medium, and high) recovery were set to no more than 15.0% CV.

#### Stability

The stability of the analyte was investigated under a variety of storage and process conditions. All stability tests were conducted in plasma, brain homogenate, and whole blood with at least three replicates at LQC and HQC levels. The impacts of light, temperature, and pH on the stability of PPIX in various biological matrices were also examined. Short- and long-term stability studies of all analytes were performed in plasma, brain homogenate, and whole blood at different storage conditions: at least 4 h at room temperature in matrix (e.g., plasma, etc.), at least 4 h in extracted processed samples at room temperature, at least 48 h after extraction in autosampler, two freeze–thaw cycles and at least 71 days at − 80 ± 15 °C. Analyte stability in biological matrices was analyzed with freshly prepared surrogate matrix QC samples. Percent difference between stability and freshly prepared QCs was also calculated. Reinjection reproducibility was also assessed within the autosampler stability period. Analytes were considered stable if the accuracy at each level was ± 15.0% of the nominal value. Additionally, the difference from corresponding comparison QC samples must be within ± 15.0%.

Short (benchtop) and long-term (4 °C) analyte stock stability and analyte working solution stability (− 80 ± 15 °C) were also determined using three replicates. Briefly, stock solution, WS 1 (LLOQ), and WS 10 (ULOQ) were compared against freshly prepared stock solution, WS 1, and WS 10. Stability was evaluated by determining the percent difference between stability and comparison samples using the mean area ratio of three replicates. Acceptance criteria were such that the percent difference was within ± 10.0% and the percent CV of three replicates was no more than ± 10.0%.

### Applications

#### PPIX levels in blood and plasma: pharmacokinetic profiling in SDT

The bioanalytical method described herein was applied to determine PPIX concentration in plasma, blood, and brain tumor of recurrent HGG patients. Plasma and blood samples were analyzed from recurrent HGG patients enrolled in an ongoing SDT trial (NCT04559685) as one of the exploratory endpoints of the trial. The protocol was approved by the Institutional Review Board (IRB) of Barrow Neurological Institute, St. Joseph’s Hospital & Medical Center (Phoenix, AZ, USA), and all patients enrolled in the trial provided written informed consent. Patients received an intravenous (IV) dose of 5-ALA (10 mg/kg), after which blood samples were taken at various time points in order to construct a pharmacokinetic profile of PPIX in blood and plasma. During the blood draws, patients were not exposed to general anesthesia. During collection, blood and plasma samples were protected from light. Plasma extraction was performed by centrifugation of blood samples for 10 min at 4 °C and 3000 rpm. Plasma and blood samples were stored at − 80 ± 15 °C until analysis. Mean peak concentrations (C_max_) and mean total exposure (AUC_0-∞_) in plasma and blood were calculated by non-compartmental analysis (NCA) using Phoenix WinNonlin version 8.3.1.5014 (Certara USA, Inc., Princeton, NJ). AUC_0-∞_ was determined by “linear up log down” rule and observed half-life was used to extrapolate to infinity.

#### PPIX levels in brain tumors: fluorescence-guided surgery

Brain tumor tissue samples (including gadolinium-contrast enhancing (ET) and non-enhancing (NET) regions) were resected from patients with newly diagnosed glioblastoma who underwent fluorescence-guided surgery according to IRB approved Ivy Brain Tumor Center Protocol 2021–15 at St. Joseph’s Hospital and Medical Center. Written informed consent for PPIX level determination in brain tumor and intraoperatively sampled whole blood and plasma was obtained from patients. All intraoperative samples were collected from patients undergoing surgery with general anesthesia. Time of resection after receiving the standard dose of Gleolan (oral, 20 mg/kg) ranged from 5.1–7.6 h. Tumor samples were rinsed with ice-cold PBS, dried on tissue paper, flash frozen in liquid nitrogen, and stored at − 80 ± 15 °C until analysis. A blood sample was also taken at the intraoperative time point, from which PPIX levels in whole blood and plasma were determined. Plasma extraction was performed as described above. Plasma and blood samples were stored at − 80 ± 15 °C until analysis.

## Results and discussion

### Method development and optimization

The bioanalytical method described in this study was developed to determine PPIX levels in human plasma, brain homogenate, and whole blood. While plasma and whole blood have been previously employed as matrices for LCMS–based method development and pharmacokinetic studies of PPIX^32,36^, this work represents the first application of human brain tissue in the development and validation of an LC–MS/MS method for PPIX analysis. Serum has also been evaluated due to its lower protein content and absence of anticoagulants, particularly in the context of assessing PPIX kinetics as a potential diagnostic biomarker for glioma^20^. Our selection of plasma (over serum) was driven by its physiological relevance for pharmacokinetic monitoring and reduced pre-analytical variability. However, all three biofluids (plasma, serum, and whole blood) are compatible for profiling of PPIX kinetics and can be selected based on specific research objectives. Two ranges, one for plasma and brain homogenate (1–2000 nM) and one for blood (10–2000 nM) were selected to account for the PPIX levels encountered in sonodynamic therapy and 5-ALA fluorescence guided tumor resection, and the endogenous levels of PPIX in blood. Several columns were tested (Phenomenex Kinetex™ Biphenyl (100 × 2.1 mm, 2.6 µm), and Phenomenex Kinetex™ F5 (100 × 2.1 mm, 2.6 µm), and Phenomenex Kinetex™ EVO C18 (50 × 2.1 mm, 1.7 µm)) along with various combinations of mobile phases to achieve an acceptable LLOQ and retention time. The Phenomenex Kinetex™ EVO C18 (50 × 2.1 mm, 1.7 µm) was selected for PPIX analysis because it achieved the most sensitive and reproducible separation of PPIX and IS. Isocratic elution was used with mobile phase A (15%) consisting of 5 mM ammonium formate and 0.1% formic acid and mobile phase B (85%) consisting of 1/1 acetonitrile/methanol and 0.1% formic acid. The total run time of the method was 3.5 min, with PPIX and IS eluting at 0.95 ± 0.3 min. Carryover was managed by using an injection volume of 3 µL with needle external wash before and after aspiration with 1/1 methanol/water rinsing solution. Source parameters for mass spectrometry were optimized with the designated mobile phases (Table 1).

**Fig. 3:**
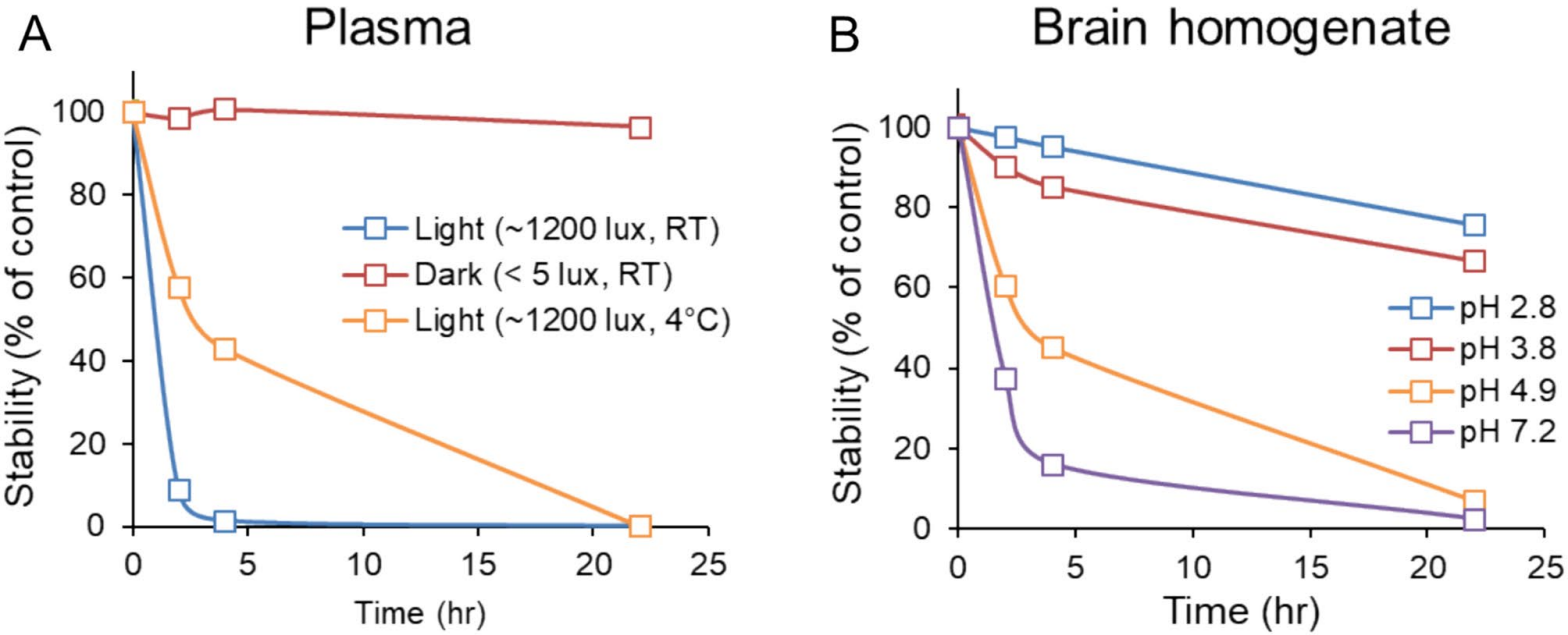
Stability experiments performed in plasma investigating the impact of light (**A**) and in brain homogenate investigating the impact of pH (**B**).

During method development, stability of PPIX in various biological matrices was investigated. First, the impact of light on PPIX levels in human plasma was investigated. PPIX plasma samples spiked with the compound were tested under three conditions: 1) on the bench exposed to light (~ 1200 lx, room temperature), 2) in a drawer protected from light (< 5 lx, room temperature), and 3) on the bench exposed to light while on wet ice (~ 1200 lx, 4 °C). Various time points were sampled, samples were analyzed for PPIX using the described bioanalytical method, and a stability curve was constructed (Fig. 3A). It was determined that PPIX samples in plasma are highly sensitive to light, with over 50% of the sample degrading in less than 2 h when exposed to light. However, when protected from light, the samples were stable at room temperature. Given the results of this experiment, all sample preparation and benchtop stability experiments were performed under low light conditions (< 5 lx). Stability was also determined in whole blood, where 64% of the total amount at time zero remained after 20 h when exposed to light (~ 1200 lx, room temperature). PPIX was stable in whole blood under dark conditions at room temperature.

PPIX levels were also degraded in brain homogenate samples when exposed to light but also when protected from light. It was hypothesized that pH adjustment of the brain homogenate could increase the PPIX stability in the sample, so an experiment was designed to test brain homogenate stability at various pH under low light conditions. Various time points were sampled, samples were analyzed for PPIX concentration using the described bioanalytical method, and a stability curve was constructed (Fig. 3B). It was found that low pH (~ 2.8) was necessary to stabilize the PPIX levels in brain homogenate for a short period of time (~ 4 h). Given the results of this experiment, all brain homogenate samples were prepared using the acidified brain homogenate procedure described in Sect. "Brain and brain tumor sample preparation". All brain tumor samples were also homogenized under acidic conditions and prepared within 1 h for analysis. Since PPIX sample acidification has been shown to result in ZnPPIX to PPIX conversion^32^, we have investigated the potential of ZnPPIX interference during sample preparation and/or its conversion to PPIX within the ion source using experimental spiking studies. The results reveal no potential interference of ZnPPIX in PPIX determination across the validated matrices (data not shown).

### Method validation results

#### Selectivity (blank check, interference check)

**Fig. 4:**
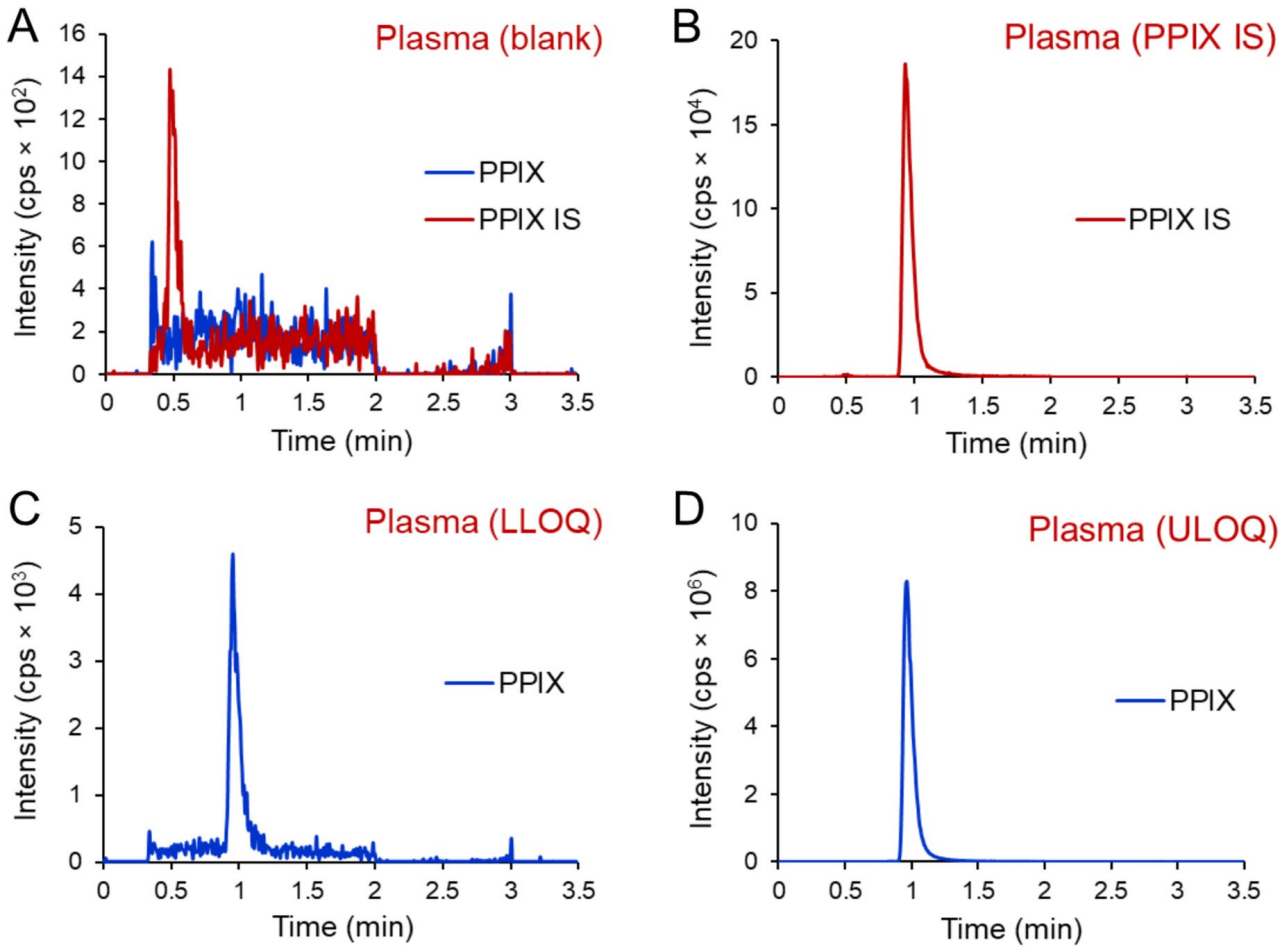
Representative ion chromatograms of blank plasma samples (**A**), plasma samples spiked with internal standard ^2^H_6_-PPIX (***m/z 569.1 → 510.1***) (**B**), plasma samples spiked with PPIX (***m/z 563.2 → 504.2***) at the LLOQ (**C**) and ULOQ (**D**).

Maximal interference at the retention times of the analyte and IS were 3.7% and 8.3% across six human plasma single donor lots and one brain tissue lot, respectively. In blood samples, the maximum interference at the retention time of the IS across 6 lots was 0.0%. Maximal interference arising from IS at the retention time of the analyte was 1.9% and maximal interference arising from the analyte at the retention time of the IS was 0.0%. Typical chromatograms of blank and IS are included in Fig. 4A and B, accordingly.

#### Calibration curve and sensitivity

For plasma and brain quantitation, a concentration range of 1–2000 nM was described using the simplest model of linear regression for the calibration curve where the average accuracy of all standards was between 98.0–101.0% with a maximum of 5.6% CV for inter-day measurements (n = 18). For blood quantitation, a concentration range of 10–2000 nM was described using a linear calibration curve where the average accuracy of all standards was between 98.9–101.0% with a maximum of 2.1% CV for inter-day measurements (n = 8). Typical chromatograms of the analyte LLOQ and ULOQ samples are included in Fig. 4C and D, accordingly.

#### Precision and accuracy

Precision and accuracy of the PPIX assay for plasma, brain homogenate, and whole blood were evaluated on an intra- and inter-assay basis. Three independent precision and accuracy runs were performed for each matrix by two chemists on different days for each matrix. Results are presented in Tables 2, 3 and 4 and meet the acceptance criteria (accuracy and percent CV were within ± 20.0% at the LLOQ and within ± 15.0% for other QC levels). Given that brain homogenate and whole blood QCs meet the pre-determined acceptance criteria, human plasma may be used as a surrogate matrix for quantitation of PPIX in brain homogenate and whole blood.

**Table 2:** Precision and accuracy results in plasma.

Analyte	QC ID	Nominal Conc. (nM)	Intra-batch (first batch)				Inter-batch			
			n	Mean Calculated Conc. (nM)	Accuracy (%)	CV (%)	n	Mean Calculated Conc. (nM)	Accuracy (%)	CV (%)
PPIX	LLOQ QC	1.00	5	1.00	100.0	3.3	15	0.965	96.5	4.9
	LQC	3.00	5	2.91	97.0	4.0	15	3.01	100.3	4.0
	MQC	60.0	5	56.3	93.8	0.9	15	59.7	99.4	4.4
	HMQC	600	5	568	94.7	1.5	15	613	102.2	5.7
	HQC	1500	5	1420	94.6	1.2	15	1520	101.3	5.0

**Table 3:** Precision and accuracy results in brain homogenate.

Analyte	QC ID	Nominal Conc. (nM)	Intra-batch (first batch)				Inter-batch			
			n	Mean Calculated conc. (nM)	Accuracy (%)	CV (%)	n	Mean Calculated conc. (nM)	Accuracy (%)	CV (%)
PPIX	LLOQ QC	1.00	5	0.951	95.1	5.9	15	1.02	102.0	10.7
	LQC	3.00	5	2.85	94.9	2.5	15	2.84	94.6	4.3
	MQC	60.0	5	54.7	91.2	0.8	15	57.9	96.4	6.5
	HMQC	600	5	553	92.1	3.4	15	571	95.1	4.6
	HQC	1500	5	1370	91.0	10.6	15	1450	96.6	7.5

**Table 4:** Precision and accuracy results in whole blood.

Analyte	QC ID	Nominal Conc. (nM)	Intra-batch (first batch)				Inter-batch			
			n	Mean Calculated conc. (nM)	Accuracy (%)	CV (%)	n	Mean Calculated conc. (nM)	Accuracy (%)	CV (%)
PPIX	LLOQ QC	10.0	5	10.9	108.9	10.4	15	11.0	110.0	6.2
	LQC	30.0	5	31.2	104.1	3.1	15	31.6	105.3	3.8
	MQC	62.9	5	59.9	95.2	2.4	15	64.4	102.4	5.8
	HMQC	624	5	592	94.9	1.9	15	625	100.1	4.4
	HQC	1520	5	1440	94.8	2.2	15	1550	101.9	6.5

#### Matrix effect

Neither ion suppression nor enhancement was observed during the matrix effect analysis. For analysis of the matrix effect in blood, it was necessary to quantify the endogenous concentration of PPIX in each lot of blood and add the amount from the working solution to the endogenous concentration for precision and accuracy evaluation. In human plasma across six lots from single donors, accuracy ranged from 89.2–106.7%, with maximal percent CV of 10.9%. In human blood across six lots from single donors, accuracy ranged from 91.3–100.0%, with maximal percent CV of 3.6%. Data are presented in Table 5. Due to the rare nature of the brain matrix, only one lot was tested, with accuracy ranging from 91.0–102.0% and maximal percent CV of 10.7%.

**Table 5:** Matrix effect test results in plasma and whole blood.

Plasma												
Analyte	Lot ID	LQC					HQC					
		Nominal Conc. (nM)	n	Mean Calculated Conc. (nM)	Accuracy (%)	CV (%)	Nominal Conc. (nM)	n	Mean Calculated Conc. (nM)	Accuracy (%)	CV (%)	
PPIX	1	3.00	3	2.86	95.3	5.4	1500	3	1600	106.7	2.4	
	2		3	2.79	92.9	10.9		3	1570	104.7	1.1	
	3		3	2.93	97.7	4.6		3	1490	99.4	2.7	
	4		3	2.68	89.2	4.8		3	1550	103.2	1.5	
	5		3	2.92	97.3	5.1		3	1500	100.1	0.8	
	6		3	2.76	92.1	10.0		3	1530	102.2	2.0	

Whole blood												
Analyte	Lot ID	Endogenous Conc. (nM)	LQC					HQC				
			Nominal Conc. (nM)	n	Mean Calculated Conc. (nM)	Accuracy (%)	CV (%)	Nominal Conc. (nM)	n	Mean Calculated Conc. (nM)	Accuracy (%)	CV (%)
PPIX	1	26.7	56.7	3	51.8	91.3	0.59	1530	3	1530	100	0.76
	2	77.7	108	3	99.2	92.1	1.9	1580	3	1540	97.5	3.6
	3	46.0	76.0	3	74.1	97.5	1.5	1550	3	1440	92.9	2.0
	4	247	277	3	275	99.3	1.6	1750	3	1660	94.9	0.29
	5	67.6	97.6	3	98.3	101	2.4	1570	3	1520	96.8	0.95
	6	84.8	115	3	109	95.0	0.57	1580	3	1460	92.4	1.7

#### Parallelism

Parallelism of the assay was evaluated for brain tumor homogenate and blood, and the percent CV of the series was determined to be 6.7% and 1.8%, respectively. The results of the evaluation are included in Table 6.

**Table 6:** Parallelism evaluation.

Dilution factor	Blood, PPIX (nM)	Brain tumor, PPIX (nM)*
1	1710	12000
2	1640	10100
5	1640	10500
10	1640	10500
20	1660	10800
Mean	1660	10780
Standard deviation	30.3	725.9
%CV	1.8	6.7

*Reported concentrations represent the PPIX concentration in the tissue sample before homogenization.

#### Recovery

Recovery for both PPIX and IS ranged between 84.2–111.3% with a maximum percent CV of 7.0% in all three matrices. Three QC levels (low, high-medium, and high) were tested in plasma, brain homogenate, and whole blood. Data are presented in Table 7.

**Table 7:** Recovery test results.

Matrix	Recovery (%)			
	LQC	HMQC	HQC	IS
Plasma	94.7	95.1	98.3	111.3
Brain homogenate	111.2	90.1	87.7	101.2
Whole blood	93.6	92.4	84.2	98.7

#### Carryover

Carryover was insignificant after injection of a 500 nM standard sample. However, at least one blank sample should be injected after samples with expected high concentrations (> 500 nM) to avoid a repeat analysis of subsequent unknown samples. Alternatively, unknown samples with calculated concentration < 20 nM that succeed samples > 500 nM will need to be repeated after the initial analysis.

#### Hemolysis effect

The hemolysis effect was not investigated due to the nature of the analyte. PPIX is an endogenous compound present in blood cells, taking part in the biosynthetic pathway of heme. The analysis of hemolytic plasma will result in additional PPIX detection arising from the rupture of red blood cells. Hemolytic samples may be analyzed based on the intentions of the study. For example, hemolyzed plasma may be analyzed if the expected concentration is relatively high, as in the case of sonodynamic therapy. A 5% hemolysis of blood cells (release of endogenous PPIX into plasma) will be negligible, based on mean endogenous PPIX levels of 58.6 ± 61.5 nM (mean ± SD) with a range of 14.5–269 nM determined in 16 blood lot samples. For application of the bioanalytical method used to screen patients for low or baseline levels of PPIX in plasma, hemolyzed plasma samples may not be accepted, since the resulting plasma concentration due to 5% hemolysis will exceed the LLOQ of the assay, causing an unacceptable interference and inaccurate results.

#### Stability

The impact of light, temperature, and pH on the stability of PPIX were evaluated before performing any of the stability tests for assay validation. In plasma, the effect of light exposure caused rapid degradation of the sample. In brain homogenate, the effect of pH had an impact on PPIX stability; the brain homogenate was therefore acidified to keep maintain sample integrity. PPIX levels in DMF (stock solutions) were also determined to be light sensitive. Stability assays were performed with three replicates in three different tubes. For analyte stability in stock and working solutions, percent difference between the mean area ratio of three replicates was calculated, as well as the percent CV of each group (Table 8). To summarize, PPIX stock solutions prepared in DMF are stable for at least 23 h under low light exposure (< 5 lx) at room temperature and 241 days at 4 °C. PPIX working solutions are stable for at least 17 h and 113 days at room temperature and − 80 ± 15 °C, respectively. The PPIX IS stock solution dissolved in DMSO showed stability for up to 432 days at 4 °C. The IS-PS showed stability at room temperature for 5 h and up to 42 days when stored at 4 °C (Table 8).

**Table 8:** Stock and working solutions stability.

Stability	Analyte				Internal standard		
	Stock solution		Working solutions		Stock solution	IS-PS	
	Room temperature	4 °C	Room temperature	− 80 °C	4 °C	Room temperature	4 °C
Period	23 h	241 days	17 h	113 days	432 days	5 h	42 days

For analyte stability in biological matrices (e.g., plasma, brain homogenate, and whole blood), surrogate QCs were prepared on the same day as the analysis and used to determine stability. The percent difference between prepared QCs and stability QCs was calculated. PPIX was stable in plasma, brain homogenate, and whole blood after two freeze–thaw cycles (with 1.5 h thawing cycle). PPIX was not stable in brain homogenate after three freeze–thaw cycles (with both 1.5 h and 2 h thawing cycles). Other evaluated stability conditions in biological matrices (extracted and non-extracted) included benchtop (short-term and room temperature), long-term (− 80 ± 15 °C), processed sample (short-term and room temperature), and autosampler (short-term, 5 °C). All benchtop stability tests were conducted under low light conditions (< 5 lx). Stability results for various periods of time are included in Tables 9 and 10.

**Table 9:** PPIX plasma and brain homogenate stability results.

Matrix	Stability	Bench Top		Freeze–Thaw		Autosampler		Processed Sample		Long-term	
	QC Level	LQC	HQC	LQC	HQC	LQC	HQC	LQC	HQC	LQC	HQC
	Nominal Conc. (nM)	3.00	1500	3.00	1500	3.00	1500	3.00	1500	3.00	1500
Plasma	Period	6 h		2 cycles		48 h		4 h		94 days	
	Mean Conc. (nM)	3.36	1720	3.22	1660	2.97	1480	3.14	1690	3.09	1580
	Accuracy (%)	112.1	114.7	107.3	110.7	99.1	98.7	104.7	112.7	103.0	105.3
	CV (%)	1.7	1.8	0.6	0.3	5.4	2.8	3.4	0.9	1.5	1.3
Brain Homogenate	Period	4 h		2 cycles		72 h		4 h		98 days	
	Mean Conc. (nM)	3.05	1460	3.18	1630	3.01	1450	3.19	1620	3.32	1580
	Accuracy (%)	101.6	97.3	106.0	108.7	100.2	96.7	106.3	108.0	110.7	105.3
	CV (%)	3.3	6.7	7.7	4.4	2.0	1.6	2.6	0.7	4.4	1.3

**Table 10:** PPIX whole blood stability results.

Matrix	Stability	Bench Top		Freeze–Thaw		Autosampler		Processed Sample		Long-term	
	QC Level	LQC	HQC	LQC	HQC	LQC	HQC	LQC	HQC	LQC	HQC
	Nominal Conc. (nM)	30.0	1530	30.0	1530	30.0	1530	30.0	1530	30.0	1530
Blood	Period	5 h		2 cycles		72 h		5 h		71 days	
	Mean Conc. (nM)	31.3	1560	31.8	1610	32.8	1600	31.5	1510	28.4	1430
	Accuracy (%)	104.3	102.0	106.0	105.2	109.3	104.6	105.0	98.7	94.7	93.5
	CV (%)	2.1	2.3	1.1	3.4	0.6	1.3	4.5	1.4	2.5	2.8

### Applications

**Fig. 5:**
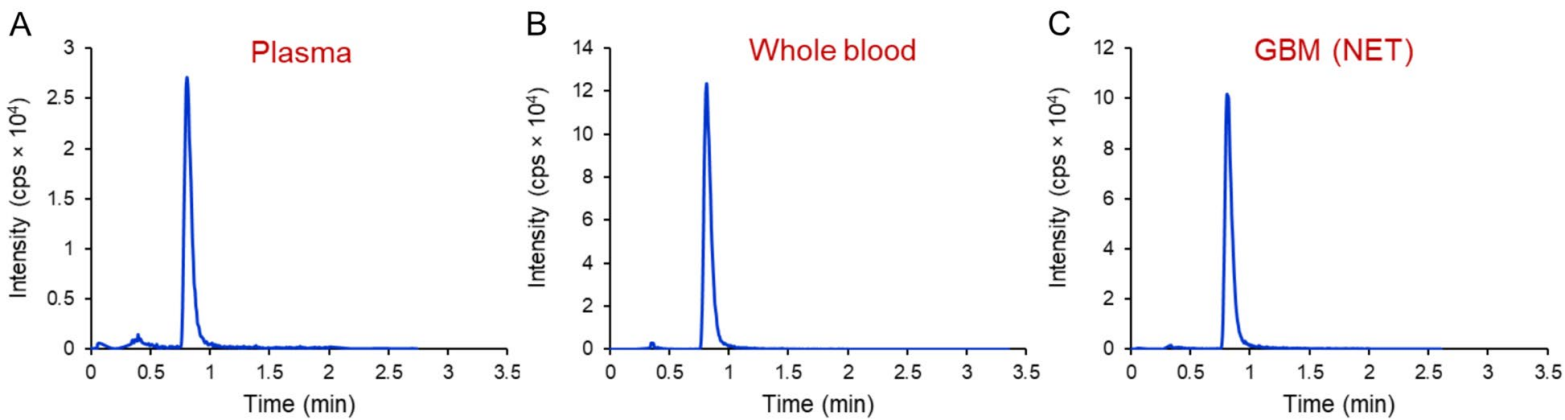
Representative ion chromatograms of PPIX in patient plasma (**A**), whole blood (**B**), and gadolinium non-enhancing brain tumor (**C**) samples collected intraoperatively 5–8 h after Gleolan administration (oral, 20 mg/kg) in the context of fluorescence-guided surgery.

The validated bioanalytical method was utilized in two different studies. The first application involved use of the method to deremine PPIX pharmacokinetic profiles in plasma and whole blood following an IV administration of 5-ALA to recurrent HGG patients enrolled in sonodynamic therapy (NCT04559685). The second application was focused on determining the PPIX levels in NET and ET regions relative to plasma and whole blood levels in newly diagnosed GBM patients. Patients involved in the second application had received Gleolan as part of the fluorescence-guided surgical resection. Example chromatograms of PPIX in patients’ plasma, whole blood, and brain tumor are shown in Fig. 5.

#### PPIX levels in blood and plasma: pharmacokinetic profiling in SDT

**Fig. 6:**
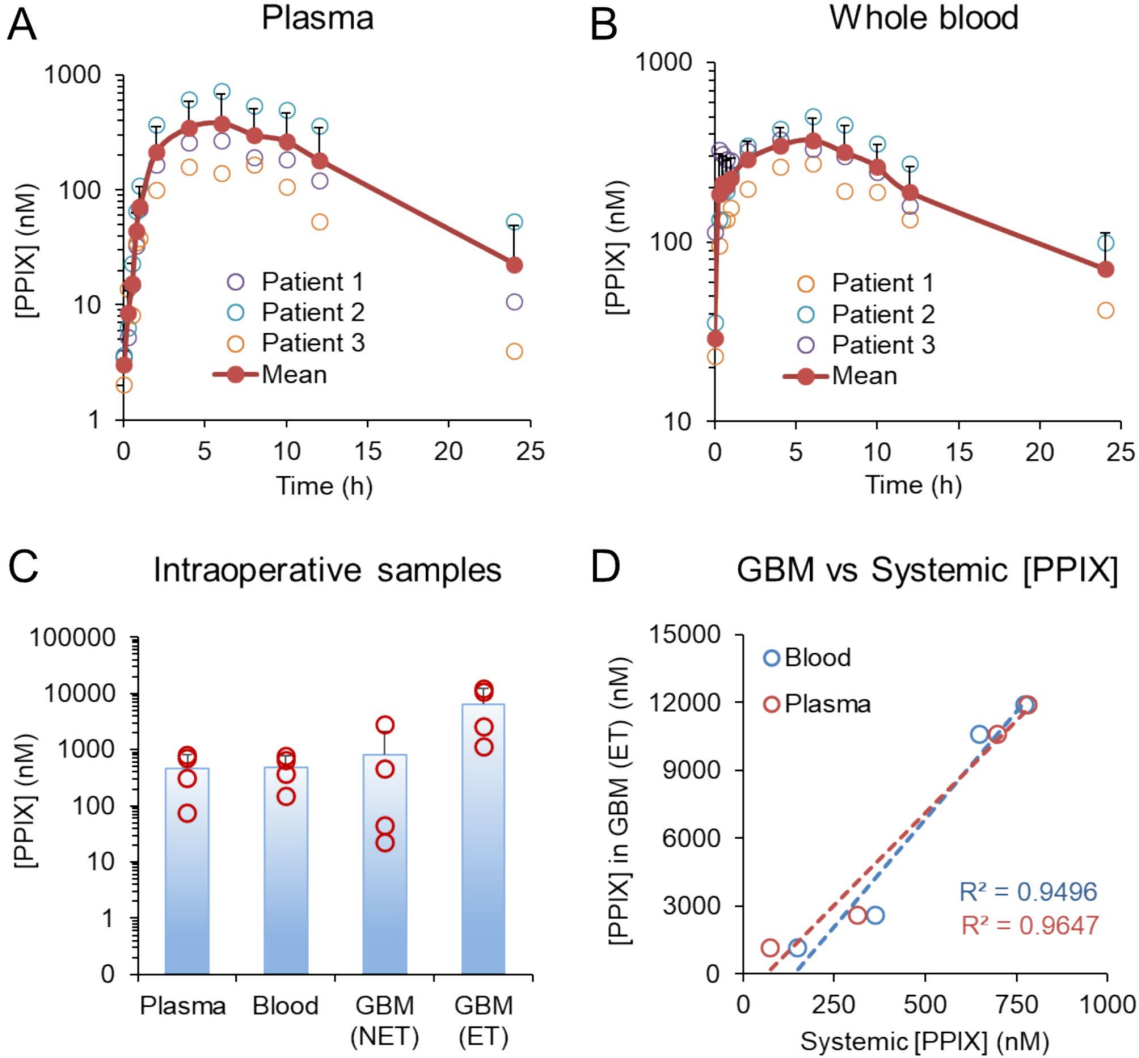
Application of the validated method in determination of PPIX levels in patient samples. Pharmacokinetic profiles of three patients enrolled in an ongoing SDT trial in plasma (**A**) and whole blood (**B**). Blood samples were taken at various time points after administration of a 10 mg/kg IV dose of 5-ALA. (**C**) Mean and individual levels of PPIX in patient plasma, whole blood, brain tumor (NET and ET regions shown) from 4 patients enrolled in clinical trial with identifier NCT05182905. Samples were obtained between 5–8 h after oral administration of 5-ALA (Gleolan, 20 mg/kg). (**D**) Individual levels of PPIX in patient intraoperative plasma and whole blood are correlated with their intraoperative brain tumor (ET) region as described in (**C**).

PPIX levels in whole blood and plasma were determined following a single 10 mg/kg IV dose of 5-ALA using the validated bioanalytical method (Fig. 6A and B). Baseline (endogenous) PPIX levels prior to 5-ALA administration averaged 3.1 ± 0.9 nM in plasma and 29.3 ± 49.3 nM in blood. After the IV dose of 5-ALA, PPIX levels in both plasma and whole blood increased rapidly, reaching peak concentrations around 6 h post-dose. The mean C_max_ (± standard deviation) values of PPIX for 3 patients were nearly identical in plasma and whole blood, measuring 385.7 (± 295.2) nM and 384.3 (± 116.4) nM, respectively. These peak values are a 126-fold increase in plasma and a 13-fold increase in blood compared to pre-5-ALA administration endogenous levels. Total PPIX baseline-unadjusted exposure (AUC_0-∞_ ± SD) of PPIX in whole blood (5371.9 ± 2098.4 h × nM) appears larger than in plasma (4167.4 ± 3398.0 h × nM). However, the values equilibrate after PPIX baseline-adjustment: 3843.7 ± 2146.3 h × nM and 4046.6 ± 3378.5 h × nM for whole blood and plasma, accordingly. The larger difference observed between baseline-adjusted and unadjusted AUC_0-∞_ values for PPIX blood exposure post 10 mg/kg IV 5-ALA administration is due to higher baseline PPIX levels in blood as compared to plasma. Previous studies have also examined both endogenous and 5-ALA-induced PPIX levels in plasma, serum, and blood, reporting significant variability across all matrices (Table 11)^17,19,20,26,32,36–40^. This variability could be attributed to differences in patient populations, as well as technical factors such as the bioanalytical methods employed and the degree of validation of the assays used for PPIX determination. For instance, in a comparable glioma patient population, a 20 mg/kg dose of 5-ALA has been shown to produce more than a tenfold variation in peak PPIX levels^20,37^. Some patient-specific variables such as sex, age, pre-existing conditions, or concomitant medications may also impact PPIX levels, further contributing to the observed variability^20^. Additional factors contributing to variability include the photo-instability of PPIX during sample collection, storage, and analysis. Furthermore, due to the low solubility of PPIX, careful selection of solvents or solvent systems that maintain PPIX stability over time is crucial when preparing stock and working solutions for calibration curves. Given that elevated PPIX levels in plasma and other biofluids are being actively investigated as potential biomarkers for various cancers^17,20,21,41^, it is critical to employ a validated, highly specific, and sensitive method for PPIX determination when developing diagnostic thresholds.

**Table 11:** Plasma, serum, and blood levels of PPIX reported in the literature.

Participants	Dose	Matrix	PPIX levels	Time	References
Endogenous					
Healthy volunteers	none	blood	> 93 nM		Hennig et al.^26^
Healthy volunteers	none	blood	~ 12 nM		Walke et al.^32^
Healthy volunteers	none	serum	11 nM		Walke et al.^20^
Healthy volunteers	none	blood	71 nM		Zhou et al.^42^
Actinic keratosis	none	plasma	~ 5 nM		Novak et al.^36^
GBM	none	blood	35 nM		Walke et al.^32^
Post-5-ALA administration					
Healthy volunteers	20 mg/kg	serum	12 nM	at 48 h	Walke et al.^20^
Healthy volunteers	20 mg/kg	serum	1032 nM	at 5 h (t_max_)	Walke et al.^20^
Healthy volunteers	1 g	plasma	2.8 nM and 0.2 nM	at 4 h and 8 h	Ota et al.^17^
Healthy volunteers	100 mg	plasma	BLQ (half samples) – 444 nM	0.1—10 h	Dalton et al.^19^
GBM	20 mg/kg	blood	101 – 114 nM	5–7 h	Walke et al.^32^
Primary HGG	20 mg/kg	serum	2742 nM	at 8.9 h (t_max_)	Walke et al.^20^
Recurrent HGG	20 mg/kg	serum	3863 nM	at 6.4 h (t_max_)	Walke et al.^20^
Gliomas (grade III and IV)	20 mg/kg	plasma	213 nM	at 7.8 h (t_max_)	Stummer et al.^37^
Bladder cancer	1 g	plasma	69.1 nM and 41.8 nM	at 4 h and 8 h	Ota et al.^17^
Proposed for PDT or diagnosis	40 mg/kg	plasma	1317 nM	at 6.7 h (t_max_)	Rick et al.^38^
Abdominal malignant conditions	30 mg/kg	plasma	994 nM	at 8.8 h (t_max_)	Webber et al.^39^
Abdominal malignant conditions	60 mg/kg	plasma	2823 nM	at 7.8 h (t_max_)	Webber et al.^39^
GI malignancy	60 mg/kg	plasma	2663 – 5859 nM	8–12 h (t_max_)	Webber et al.^40^

#### PPIX levels in brain tumors: fluorescence-guided surgery

The validated bioanalytical method for PPIX quantification was also employed to measure PPIX levels in patient GBM tumor regions (ET and NET), whole blood, and plasma samples collected intraoperatively 5.1–7.6 h after Gleolan administration (oral, 20 mg/kg) during fluorescence-guided surgery (Fig. 6C). Similar PPIX concentrations were observed in plasma and blood with mean PPIX levels of 467 ± 331 nM and 484 ± 281 nM (n = 4), respectively. Given the reported 60% bioavailability of the oral formulation of 5-ALA^19^, higher PPIX levels observed with the 20 mg/kg oral Gleolan dose compared to the 10 mg/kg IV dose, as described in Sect. "PPIX levels in blood and plasma: pharmacokinetic profiling in SDT", were anticipated. However, these findings contrast with previously published data, which report peak PPIX concentrations ranging from as low as ~ 100 nM^32^ to as high as 3,863 nM^20^ in a comparable patient population following 20 mg/kg oral 5-ALA administration (Table 11). In another study, erratic PPIX levels were noted, with nearly half of the patients showing levels below the limit of quantification (BLQ) after 5-ALA administration^19^. These diverse results emphasize the importance of using a validated, highly specific bioanalytical method when determining PPIX levels in biofluids.

In GBM tumor, the mean PPIX levels were 815 ± 1290 nM in NET regions and 6560 ± 5480 nM in ET regions. The substantial variability in PPIX concentrations within brain tumors is likely due to the heterogeneous distribution of tumor cells in both ET and NET areas. Similar variability has been reported in previous studies on tumor tissue PPIX levels^43,44^. Across all patients, PPIX concentrations were consistently higher in the ET region compared to the NET region, which is expected as the ET region is the tumor core, characterized by a higher density of tumor cells, while the NET region represents the infiltrative edge^45^. Importantly, PPIX levels in plasma and whole blood in all four patients analyzed showed a positive correlation with their intratumoral PPIX concentrations in the ET region (Fig. 6D). Although the sample size is small, these preliminary findings suggest that systemic PPIX measurements may serve as a potential correlate for intratumoral PPIX exposure. Since PPIX levels arise from exogenous 5-ALA administration, the observed systemic levels reflect both biosynthesis and tissue distribution dynamics, which are complex and multifactorial^46^. Therefore, while the data show a correlation between systemic and tumor PPIX levels in individual patients, high systemic levels may not exclusively reflect tumor-specific uptake or production. Comparable levels of PPIX with significant variability have been previously reported for the infiltrative grade IV tumors (1.0 ± 1.0 µM) and GBM tissue with high tumor cell density (5.8 ± 4.8 µM)^44^, likely representing the NET and ET regions, respectively. Additionally, several studies have noted a positive correlation between Gd-enhancement and PPIX fluorescence in glioma tissues^43,47^. Relatively lower average PPIX levels have been detected in glioma tissue (2.87 µM), which may be attributed to the inclusion of both low- and high-grade glioma patients in the study^43^. The same study reported significantly lower PPIX levels in grade IV tumors with necrotic areas (0.5 ± 0.5 µM) and grade III tumors (0.2 ± 0.4 µM)^44^. While lower PPIX concentrations are expected in lower-grade tumors, other studies have reported average PPIX levels exceeding 15 µM in grade III brain tumors^47,48^. PPIX fluorescence has also been detected in recurrent malignant gliomas and metastatic brain tumors^49^. Overall, tissues showing high fluorescence consistently exhibited high PPIX concentrations, regardless of tumor grade, reinforcing the role of PPIX in tissue fluorescence^47,48^. Importantly, PPIX levels in non-neoplastic brain tissue were either undetectable or very low^44,50^, underscoring the critical role of tumor cells in the uptake, generation, and accumulation of PPIX following 5-ALA administration^43,51^.

Further research is required to better understand the heterogeneous distribution of PPIX in different brain tumor regions and brain tumor types. The validated method used here provides direct and reproducible measurements, offering confidence in the accuracy of the PPIX levels measured within tumor tissue as we investigate tumor heterogeneity.

## Conclusion

A sensitive, specific, and rapid LC–MS/MS method was developed and validated for the determination of PPIX in human plasma, blood, and brain tumor. Human plasma was used as a surrogate matrix for the determination of PPIX levels in brain tumor and blood samples. The present method offers high sensitivity with minimal sample preparation (protein precipitation with methanol). While several LCMS-based methods have been previously reported, none were validated, and many were either qualitative or had unknown sensitivity^25,41,52^. The reported quantitative LC–MS/MS methods for PPIX in human biospecimens are at least 5 times less sensitive and require more extensive sample preparation^20,32,53^. A paper by Novak et al. was the only publication to report two LCMS-based bioanalytical methods that were employed to support clinical pharmacokinetics of a 5-ALA nanoemulsion gel and PPIX during the PDT of patients with actinic keratosis^36^. The methods were developed and internally validated for PPIX quantitation in patients’ plasma within 1–100 ng/mL (1.78–178 nM) concentration range utilizing guidelines provided in González et al.^54^. However, the paper has provided plasma precision and accuracy results only for one method, and did not report any other critical validation test results, such as calibration curve linearity, selectivity, specificity, carryover, matrix effects, recovery, and analyte stability, which limits its utility and its establishment in broader research settings. In contrast, our validated method incorporates comprehensive reports on critical validation test components. Compared to reported method in Novak et al.^36^, our method features (1) wider dynamic range – 2000-fold for plasma and brain tumor samples, and 200-fold for blood – adequate for the PPIX levels typically encountered in these specimens, (2) higher sensitivity, (3) shorter run time, (4) lower sample volume, (5) no acidification step in plasma or blood sample preparation step, mitigating potential ZnPPIX → PPIX conversion^32^, and (6) validation across three biomatrices (plasma, blood, and brain tumor). Hence, to our knowledge, this is the first report to describe full validation of an LC–MS/MS method for the quantitation of PPIX in human plasma, brain tumor, and blood specimens, in line with ICH guidelines^28^. The method has been successfully applied to measure PPIX pharmacokinetics in the SDT clinical trial and has provided preliminary insights into PPIX distribution in brain tumors. Positive correlations were observed between systemic PPIX levels in plasma and blood and intratumoral (ET) PPIX concentrations in four patients undergoing FGS. Due to the small cohort size (3 patients with SDT and 4 patients with FGS), no correlative analyses between patient-specific variables and 5-ALA-induced PPIX levels were conducted. We are currently collecting additional data from a larger cohort, which will allow us to explore these relationships more rigorously in the future. Further research will also be needed to fully understand the spatial distribution of PPIX within brain tumor tissues.

## Supplementary Information

Supplementary Information.

## Data Availability

The raw data related to Tables 2, 3, 4 and 5, 7, and Fig. 6 are available in a Supplementary Information file.
